# Factor structure and psychometric properties of a Persian translation of the Epworth Sleepiness Scale for Children and Adolescents

**DOI:** 10.15171/hpp.2018.27

**Published:** 2018-07-07

**Authors:** Vida Imani, Chung-Ying Lin, Shabnam Jalilolgadr, Amir H. Pakpour

**Affiliations:** ^1^Department of Pediatrics, Children Growth Research Center, Qazvin University of Medical Sciences, Qazvin, Iran; ^2^Department of Rehabilitation Sciences, the Hong Kong Polytechnic University, Hung Hom, Hong Kong; ^3^Social Determinants of Health Research Center, Qazvin University of Medical Sciences, Qazvin, Iran; ^4^Department of Nursing, School of Health and Welfare, Jönköping University, Jönköping, Sweden

**Keywords:** Adolescence, Child, Daytime sleepiness, Factor analysis, Rasch analysis

## Abstract

**Background:** Given the high prevalence of excessive daytime disorder (EDS) among children and adolescents, daytime sleepiness should be effectively measured for them to design appropriate intervention program. However, the commonly used instrument Epworth Sleepiness Scale for Children and Adolescents (ESS-CHAD) has little information in its psychometric properties. This study aimed to apply 2 different test theories to examine the psychometric properties of the Persian ESS-CHAD among a large sample of Iranian adolescents and children.

**Methods:** In this methodological study, participants from 8 high schools (n=1371; 700 males), in Qazvin, Iran, completed the ESS-CHAD, a background information sheet, and Insomnia Severity Index (ISI). The ESS-CHAD was translated by using a forward-backward translation method.Two weeks later, the participants completed the ESS-CHAD again. Internal consistency using Cronbach’s alpha, test-retest reliability using intraclass correlation coefficient (ICC), regression analysis testing the correlation between ESS-CHAD and ISI, Confirmatory factor analysis (CFA)with measurement invariance, Rasch analysis with differential item functioning (DIF), and latent class analysis (LCA) were used to examine the psychometric properties of the ESS-CHAD.

**Results: ** The internal consistency (α=0.79), test-retest reliability (ICC=0.84), regression findings(β=0.39, P<0.001), CFA (comparative fit index [CFI])=0.974, root-mean square error of approximation [RMSEA]=0.040), supported measurement invariance (∆CFI=-0.009 to 0.007,∆RMSEA=-0.009 to 0.001), Rasch analysis (infit mean square=0.88 to 1.31, outfit mean square=0.68 to 1.19), and no substantial DIF (DIF contrast=-0.43 to 0.38) all indicated that ESSCHAD is a reliable and valid instrument. The LCA further classified the sample into 2 distinctclasses.

** Conclusion:** Persian ESS-CHAD could be used to assess daytime sleepiness for adolescents who are speaking Persian.

## Introduction

There is an increasing attention on daytime sleepiness among adolescents worldwide, especially the estimated prevalence of children and adolescents suffering from excessive daytime disorder (EDS) is more than 40%.^1^ EDS has been associated with a number of problems including poor sleep quality, low school performance and learning, mood impairment, accident injury and other adverse consequences.^2^ Hence, using a validated instrument to assess EDS is critical for healthcare providers. However, only a limited number of objective and subjective measures have been developed to assess EDS among populations, including multiple sleep latency test, maintenance of wakefulness test, Karolinska sleepiness scale and Epworth Sleepiness Scale (ESS).^3^ Among the instruments, the ESS is one the most widely used measure for assessing subject’s average sleep propensity in daily life. The ESS is a simple and short self-reported measure of daytime sleepiness that has originally developed for adults in 1990 by Johns.^4^ The psychometric properties of the ESS have been confirmed across various populations, cultures, and clinical conditions.^5-7^ Moreover, the ESS for Children and Adolescents (ESS-CHAD) has been recently developed.^8^ The ESS-CHAD is a modified version of ESS with replacing some appropriate items for children and adolescents. However, the information regarding the psychometric properties of the ESS-CHAD is insufficient in the current literature because only Janssen et al^9^ has reported such information.

In order to comprehensively assessing the psychometric properties of the ESS-CHAD, we strongly recommend using different test theories to portray the reliability and validity of the ESS-CHAD. Moreover, we agree with the statement made by Lin et al^10^ that “a sound instrument needs to be tested using different statistical methods across different populations, because the nature of scientific inquiry is to accumulate evidence using different methods.” Currently, 2 types of test theory, Item Response Theory (IRT) and Classical Test Theory (CTT), can be applied to assess psychometric properties of a questionnaire. CTT focuses on total summed scores without weighting or standardization; IRT separates the characteristics from questionnaires and respondents, and uses the probabilities to identify the item difficulty (i.e., whether the item makes the respondents to report low or high scores regardless the respondents’ ability) and person ability (i.e., whether the person has the ability to get low or high scores regardless the items’ difficulty). Also, Rasch analysis is a type of the IRT that it assumes a given scale is unidimensional and a person’s response is to each item is independent of their response on other items.^11^

The validity and reliability information of the ESS-CHAD in healthy adolescent populations is scarce in the literature. Thus, evaluation of these properties in a new context and culture is strongly recommended,^12^ and researchers can subsequently understand the structural validity of the ESS-CHAD.^12^ The aim of the study was therefore to assess psychometric properties of the ESS-CHAD among Iranian adolescents using both CTT and Rasch approaches.

## Materials and Methods

### Participants and procedure

In this cross-sectional study, participants were recruited from high schools from Qazvin city (a city near to Tehran), Iran. Using a multistage sampling procedure with a stratification of gender, 8 high schools (four boys’ schools and four girls’ schools) were randomly selected from a list of 57 high schools in Qazvin city. The sampling procedure was stratified by gender because all high schools in Iran are gender specific. Two classes were then selected randomly from each school and all student in the class were invited to participate in the study. Inclusion criteria were: (1) students aged 12-19 years, (2) students were able to understand Persian language, and (3) students had provided an informed consent with a parental consent if necessary. Adolescents with chronic disability/diseases and cognitive impairment were excluded from the study. All participants gave their informed consents. Parental consent was given if participants were younger than 18 years of age.

### Translation Procedure 

The permission to translate was granted by MAPI Research Trust (Lyon, France). Translation procedure was performed in a standardized process that includes several steps adhering to international guidelines.^13^ In the first step, the English version the ESS-CHAD was translated into Persian by 2 bilingual translators who were native Persian speakers. The translated versions were compared and synthesized into a unified Persian version in a session with the translators and a project manager. The interim Persian version was then translated back into English language by 2 bilingual English translators who were not aware of the original ESS-CHAD. All translated versions were reviewed and consolidated in a session by several experts, including psychologist, pediatrics, nurse, psychometrist and sleep specialist. The prefinal Persian version of ESS-CHAD was piloted on as ample of 33 adolescents (18 girls and 15 boys, mean age = 15.10). Additional changes were made on the prefinal version based on information collected form the adolescents. The final Persian version of the ESS-CHAD was then administered on 1371 adolescents.

### Instruments

Sociodemographic information was collected using a self-administered questionnaire, which included items on age, gender, parental education, smoking habits and living condition.

### Insomnia Severity Index 

The Insomnia Severity Index (ISI) is a 7-item self-report measure assessing the severity and effects of insomnia during the past month. All items are rated on a 5-point Likert scale ranging from 0 (no problem) to 4 (very severe problem). All responses are summed up to obtain a total score ranging from (0-28) with 5 subscores: 0-7 (absence of insomnia), 8-14 (sub-threshold insomnia), 15-21 (moderate insomnia), and 22-28 (severe insomnia). The ISI has been translated into several languages including Persian.^14^ The Persian version of the ISI has been found to be valid and reliable among Iranian patients with insomnia.

### The Epworth Sleepiness Scale for Children and Adolescents 

The ESS-CHAD is an 8-items questionnaire assessing the severity of daytime sleepiness among children and adolescents.^8^ All items are rated on a 4-type Likert scale ranging from 0 (would never doze or sleep) to 3 (high chance of dozing or sleeping). The ESS-CHAD total score could be computed by summing responses to all 8 items ranging from 0 to 24 with higher scores indicating greater sleepiness.

### Data Analysis

Data were presented using mean (SD), median (min-max) for the numeric normal and non-normal variables respectively and frequency (percent) for categorical variables. Both CTT and Rasch methods ware used to assess psychometric properties of the ESS-CHAD. As to the CTT, ceiling/floor effects (with a percentage <20% suggesting acceptable), internal consistency (with a Cronbach’s alpha >0.7 indicating acceptable), test-retest reliability (was measured in all adolescents across 2 weeks using an intraclass correlation coefficient (ICC) value >0.7 suggesting acceptable), corrected item-total correlation (with a value >0.4 indicating acceptable), standard error of measurement (with a smaller value suggesting better), average variance extracted (AVE; with a value >0.5 indicating acceptable), and composite reliability (with a value >0.6 suggesting acceptable) were conducted.^15^

Content and face validity were also assessed for the ESS-CHAD. Ten experts were chosen from practical fields to estimate content validity of the Persian ESS-CHAD according to the Streiner and Norman’s recommendations.^16^ All experts had experiences on sleep medicine and working on adolescents for at least 10 years. The experts were asked to evaluate the items and related responses in terms of wording, grammar, item location and scaling of the questionnaire. Content validity index (CVI) and the content validity ratio (CVR), were then calculated. Regarding faced validity, 10 adolescents were asked to indicate their opinions about each item based on its importance. The impact score (frequency × importance) was then calculated to indicate that the parentage of the adolescents who identified the item as important or quite important. An impact score of 1.5 or greater were considered satisfactory.^17^

In addition, confirmatory factor analysis (CFA) was applied to assess construct validity and multiple regression model (with dependent variable as ESS-CHAD, independent variable as ISI, and confounders as age, gender, and father’s education) to examine criterion-related validity.

The CFA was performed using full-information maximum likelihood estimator. Model fit was assessed using several indices including: a nonsignificant chi square, the root mean square error of approximation (RMSEA; <0.08 indicating acceptable), comparative fit index (CFI; >0.9 indicating acceptable), Tucker–Lewis index (TLI; >0.9 indicating acceptable), and standardized root mean square residual (SRMR; <0.08 indicating acceptable). Multigroup CFA was further performed to assess factorial invariance across the study subgroups (i.e. gender, living condition, and insomnia condition). There models were tested in the factorial invariance: model 1 (configural invariance: no equality constraints), model 2 (metric invariance: constraining factor loading to be equal across study subgroups) and model 3 (scalar invariance: imposing equality constrains on factor loadings and item intercepts). To evaluate the fit of the different models, ΔCFI <-0.01, ΔRMSEA <0.015 and ΔSRMR <0.01 were used.^18^

Rasch model was applied to estimate item difficulty, unidimensionality, item validity, item and person separation reliabilities, and item and person separation indices. The information-weighted fit statistic (infit) mean square (MnSq) and outlier-sensitive fit statistic (outfit) MnSq were used with a range between 0.5-1.5 suggesting acceptable fit. The item reliability and person reliability of the ESS-CHAD was also assessed using Rasch model with values greater than 0.7 are considered to be acceptable. Moreover, item and person separation indices were calculated to assess whether the items are able to define distinct levels of daytime sleepiness, and values of greater than 2 indicate acceptable for item and person separation indices. Differential item functioning (DIF) was employed to identify whether the subgroups of the adolescents (i.e. gender, living condition and insomnia condition) interpret the items of the ESS-CHAD differently, and values lower than 0.5 logits indicate no substantial DIF.

Finally, latent class analysis (LCA) was conducted to identify potential subgroups of adolescents according to the ESS-CHAD scores. The LCA is a model-based clustering method that tries to find latents or clusters of a given sample in multivariate data.^17^ The number of classes was determined using several indices, Bayesian Information Criterion (BIC) and Akaike’s Information Criterion (AIC), with lower values indicating better fitting model. Classification accuracy (entropy; higher values indicating better classification accuracy) and the adjusted Lo-Mendel-Rubin loglikelihood ratio test (a significant result indicates that the current is better fit to that data than a model with 1 less class) were also used to determine the classified latents. The differences across the groups were compared using chi-square test or independent *t* test. The CTT analyses, Rasch model and LCA were conducted using SPSS version 24 (IBM, Somers, NY), WINSTEP 4.01 (Winsteps, Chicago, IL) and MPLUS (Los Angeles, CA), respectively.

### Sample Size 

Sample size was estimated based on the CFA and Rasch analysis. According to the literature, a minimum of 200 participants were needed to run CFA.^19,20^ In addition, The ‘rule of thumb’ for sample size requirements suggests that 20 participants are required per item in a factor analysis. On the other hand, studies recommended to recruit equal or gather than 250 participants for Rasch analysis. Therefore, our sample size (n = 1371) was sufficient to conduct all analyses.

## Results

### Characteristics of the Sample

Of 1525 approached adolescents, 85 (5.6%) adolescents declined to participate and 69 (4.5%) adolescents were not eligible. In total, 1371 adolescents completed the study. The mean age was 15.5 years (SD 1.6; range 12–18) and 671 (48.9%) were girls. The mean age of educational attainment for father and mother were 8.7 years (SD 2.1; range 0–21) and 7.5 years (SD 2.6; range 0-21), respectively. Table 1 shows adolescent characteristics. Three hundred six adolescents reported to have insomnia (22.3%), according to cutoff of 10 for the ISI.

### Content and Face Validity 

The result of quantitative content validity showed that the CVI and CVR were 0.93 and 0.88, respectively. Moreover, 70% of the expressed that the-four-point response category is suitable. The results of impact score indicated that all items were preserved for the following steps (impact scores ranged from 1.6 to 5).

Both CTT and Rasch models showed acceptable psychometric properties (Table 2). The results of the CFA showed that the one-factor model provided acceptable fit to the data. All factor loadings were higher than 0.60. The unidimensionality of the ESS-CHAD was assessed also using the Rasch model. The results showed acceptable model fit for all 8 items of the ESS-CHAD. The DIF analysis did not reveal any differences between the adolescents’ subgroups (gender, living condition and insomnia) in all 8 items of the ESS-CHAD (DIF<0.5).

All items were scientifically correlated with the scale (r >0.50, *P *< 0.001), corrected for overlap. The test–retest reliability for the 2-week interval was 0.78 for total score (Table 2).

There were no substantial floor and ceiling effects for the ESS-CHAD (Table 3). Both AVE and CR exceeded the cut-off values indicating convergent validity of the ESS-CHAD. As expected, internal consistency was acceptable in both CTT (Cronbach’s α = 0.79) and Rasch models (person separation reliability = 0.78; item separation reliability = 1.0).

In addition, tests of factorial invariance showed that all items of the ESS-CHAD were perceived similarly across boys’ and girls’ groups, adolescents with private room and those without private room, and adolescents with insomnia and those without insomnia (Table 4). A stepwise multiple regression showed that older adolescents and higher score in ISIS were associated with daytime sleepiness (Table 5).

A series of 2- to 4-class models were tested using LCA to find out subgroups of adolescents with daytime sleepiness. The fit indices are shown in Table 6. The LMRT revealed chi-square difference between 2-class and one-class models to be statistically significant. Despite the fact that 4-class model yielded the lowest BIC, AIC and SSABIC but the LMRT was not significant indicating that the 4-class solution did not provide superior fit to the 2-class solution (Table 6). There 2 distinct group of adolescents in terms of their scores on daytime sleepiness were revealed: class 1, a low-risk day time sleepiness (n = 595) and class 2, a high-risk of daytime sleepiness (n = 771). Finally, the dominant latent classes were compared in terms of age, gender, ISI, father education and ESS-CHAD total score. The results showed that adolescents at high-risk of daytime sleepiness were older, female, higher scores in ISI, having fathers with high education and high ESS-CHAD score (Table 7).

## Discussion

The present study was conducted to assess validity and reliability of the ESS-CHAD using both CTT and Rasch measurement models. The results of the study showed that the ESS-CHAD had unidimensional structure and factorial invariance across gender, insomnia and living condition groups. In addition, the results of the study supported internal consistency, reproducibility, convergent validity and construct validity of the Persian ESS-CHAD. In this study, the LCA was applied to find potential classes in adolescents according to the ESS-CHAD for the first time. Using LCA, we have identified 2 different classes of daytime sleepiness namely ‘low-risk daytime sleepiness’ and ‘high-risk daytime sleepiness’. There results showed that although it has been recommended 5 categories for the ESS in adults (lower normal, higher normal, mild excessive, moderate excessive and sever excessive daytime sleepiness),^4^ it seems that children and adolescents may not be able to classified into 5 groups. The results of the study contribute to the literature on adolescent’s sleep patterns by identifying the relevant classes on daytime sleepiness.

The mean item distribution of the ESS-CHAD was similar in previous studies on adolescents using adult ESS.^22-24^ As results of the study showed, ceiling and floor effects of the ESS-CHAD were minimal indicating measurement range of ESS-CHAD covered adequately the concept of daytime sleepiness. Therefore, ESS-CHAD is able to detect changes in daytime sleepiness among apparently healthy adolescents. Moreover, the ESS-CHAD was found to be stable across a 2-week period (ICC >0.70), and the result is comparable to that of a previous test-retest ESS-CHAD study performed on adolescents in Australia.^9^ The internal consistency of the ESS-CHAD, as assessed by Cronbach’s alpha, was also comparable with the one reported in Australia.

The present study confirmed the one-factor structure of the ESS-CHAS using both CFA and Rasch methods. Therefore, all items of the ESS-CHAS could be conceptualized as daytime sleepiness. Janssen et al^9^ revealed that all items of the ESS-CHAS could be categorized into single construct. Moreover, the results are consistent with the adult version of the ESS.^21-27^ To the best of our knowledge, DIF has never been investigated for the ESS-CHAD. Our DIF analysis extended the current knowledge that there were no any significant differences on adolescents’ perceptions on gender, living condition and insomnia condition groups. Therefore, all items of the Persian ESS-CHAD did not show any significant statistical properties across adolescents’ subgroups. Similar results were found for Adult ESS for gender and age. However, the results should interpret with caution because we did not assess DIF for clinical sample and other socio demographic variables.^25,28^

The results of the LCA found that the patterns of the daytime sleepiness can be identified into 2 classes. The differences between the 2 classes of daytime sleepiness were captured by age, gender, ISI score, father’s education and ESS-CHAD total score. In line with our study, previous studies have also found that older adolescents suffer from higher level of sleepiness.^1,29^ The reason maybe that adolescents do not prioritize their sleep over social and leisure activities.^30^ Furthermore, being a female adolescent was associated with higher scores on ESS-CHAD.^31^ One of potential reasons could be polycystic ovarian syndrome which is common among adolescent’s girls.^1,32^ However, our study did not measure the polycystic ovarian syndrome and future studies are warranted to test our postulation. In addition to this, both girls and boys perceived all ESS-CHAD items similarly, based on results of DIF and factorial invariance across gender.

According to our results, it is not surprising that insomnia increases the chance of daytime sleepiness among adolescents. Nevertheless, there is still room for studying mediators of insomnia and daytime sleepiness among adolescents.^33^

The study has some limitations that should be acknowledged. First, the data were collected from high schools in Qazvin. Therefore, the representativeness of our sample might not be able to generalize to all Iranian adolescents. Second, we were unable to recruit all adolescents in the same time of a day. Therefore, this might affect adolescents’ responses regarding their daytime sleepiness and insomnia experience. Third, confounding factors (e.g. psychiatric problems) were not assessed among adolescents. Furfure studies should assess predictive validity of ESS-CHAD for psychiatric problems and other clinical issues. Finally, objective evaluation of sleepiness did not perform. Therefore, we recommended that future studies should use objective sleepiness measure to explore its associations with the self-reported ESS-CHAD and defining an acceptable cutoff point.

## Conclusions

The results of the study showed that Persian version of ESS-CHAD had a good internal consistency, stability over short time, acceptable measurement range, unidimensional factor structure, and measurement invariance across factorial invariance across gender, livening condition and insomnia groups. Persian language is not only as a formal language of Iran but also one of dominate language in the middle east. Therefore, Persian version the ESS-CHAD could be used to assess daytime sleepiness for adolescents who are speaking Persian.

## Ethical approval

All procedures performed in studies involving human participants were in accordance with the ethical standards of the institutional and/or national research committee and with the 1964 Helsinki Declaration and its later amendments or comparable ethical standards. A permission was obtained from ministry of Education in the Qazvin region before the study commenced. This study was approved by the authors’ institutional review board (approved protocol No. IR.QUMS.REC.1396.409) and participant consent was provided before any data collection activities.

## Competing interests

The authors declare that they have no competing interests.

## Authors’ contributions

AHP performed analyses. VI collected the data. AHP, VI and C-YL drafted the manuscript. SJ supervised entire study and All authors have reviewed and edited the manuscript. All authors have read and approved the final version of the manuscript and agree with the order of presentation of the authors.

## Acknowledgments

The authors wish to express their sincere gratitude to all those who participated in this study.

**Table 1:** Participants characteristics (N=1371)

	**Mean (SD) or No. (%)**
Age (y)	15.5 (1.6)
Gender (male)	700 (51.1)
Fathers’ educational year	8.7 (2.1)
Mothers’ educational year	7.5 (2.6)
Current smoker (yes)	280 (20.4)
Living condition: private room (yes)	953 (69.5)

**Table 2:** Psychometric properties of the Epworth Sleepiness Scale for Children in item level

**Item #**	**Item score**	**Item score**	**Analyses from classical test theory**			**Analyses from Rasch**					
	**Median**	**Mean (SD)**	**Factor loading** ^a^	**Item-total correlation**	**Test-retest** ^b^	**Infit MnSq**	**Outfit MnSq**	**Difficulty**	**DIF contrast across gender** ^cd^	**DIF contrast across living condition** ^ce^	**DIF contrast across insomnia condition** ^cfg^
ESS-CHAD1	1	0.75 (0.89)	0.66	0.65	0.73	0.89	0.99	0.09	-0.06	0.04	-0.13
ESS- CHAD2	1	0.72 (1.17)	0.75	0.53	0.77	1.31	1.19	0.15	0.38	-0.07	-0.27
ESS- CHAD3	0	0.70 (0.91)	0.65	0.70	0.78	0.88	0.88	0.22	0.18	0.10	-0.43
ESS- CHAD4	1	1.12 (1.06)	0.62	0.76	0.80	1.14	1.13	-0.58	-0.06	0.01	-0.11
ESS- CHAD5	2	1.84 (1.12)	0.79	0.66	0.86	1.06	1.10	-1.24	-0.06	0.01	0.01
ESS- CHAD6	0	0.19 (0.48)	0.75	0.75	0.82	0.90	0.68	1.36	0.08	0.24	-0.09
ESS- CHAD7	1	1.15 (1.08)	0.83	0.65	0.74	0.99	0.94	-0.63	-0.08	-0.03	0.04
ESS- CHAD8	0	0.52 (0.64)	0.78	0.71	0.72	1.10	1.06	0.62	-0.31	-0.11	-0.06

ESS-CHAD= Epworth Sleepiness Scale for Children and Adolescents, Total EDSS-CHAD score: mean (SD)= 7.91 (4.35) ^a^ Based on the first-order confirmatory factor analysis. ^b^ Using intraclass correlation coefficient (ICC). ^c^ DIF contrast > 0.5 indicates substantial DIF. ^d^ DIF contrast across gender=Difficulty for females-Difficulty for males. ^e^ DIF contrast across living condition= Difficulty for participants with private bedroom - Difficulty for participants without private bedroom MnSq=mean square error; DIF=differential item functioning. ^f^DIF contrast across insomnia condition= Difficulty for participants with insomnia - Difficulty for participants without insomnia. ^g^Based on the cutoff score of 10 in the Insomnia Severity Index.

**Table 3:** Psychometric properties of the Epworth Sleepiness Scale for Children in scale level

**Psychometric testing**	**Value**	**Suggested cutoff**
Ceiling effects (%)	1.8	<20
Floor effects (%)	0.8	<20
Internal consistency (Cronbach’s α)	0.79	>0.7
CFA		
χ^2^ (*df*)	28.84 (20)^a^	Nonsignificant
Comparative fit index	0.974	>0.9
Tucker-Lewis index	0.919	>0.9
Root-mean square error of approximation	0.040	<0.08
Standardized root mean square residual	0.021	<0.08
Average variance extracted	0.54	>0.5
Composite reliability	0.90	>0.6
Standard error of measurement	1.99	The smaller the better
Item separation reliability from Rasch	1.00	>0.7
Item separation index from Rasch	21.55	>2
Person separation reliability from Rasch	0.78	>0.7
Person separation index from Rasch	2.53	>2
Test-retest reliability by ICCs	0.84	>0.4

Abbreviations: ICC, intraclass correlation coefficient. ^a^ *P* < 0.001.

**Table 4:** Measurement invariance across gender and across living condition on Epworth Sleepiness Scale for Children using confirmatory factor analysis

**Model and comparisons**	**Fit statistics**							
	**χ** ^ 2 ^ ** (df)**	**∆χ** ^ 2 ^ **(∆df)**	**CFI**	**∆CFI**	**SRMR**	**∆SRMR**	**RMSEA**	**∆RMSEA**
Gender								
M1: Configural	82.71 (20)^a^		0.919		0.043		0.051	
M2: Plus all loadings constrained	94.31 (28)^a^		0.923		0.040		0.052	
M3: Plus all intercepts constrained	107.09 (36)^a^		0.930		0.036		0.050	
M2−M1		11.6 (8)		0.004		-0.003		0.001
M3−M2		12.78 (8)		0.007		-0.004		-0.002
Living condition on ESS								
M1: Configural	118.52 (20)^a^		0.965		0.032		0.043	
M2: Plus all loadings constrained	130.39 (28)^a^		0.956		0.034		0.044	
M3: Plus all intercepts constrained	151.14 (36)^a^		0.964		0.030		0.035	
M2−M1		11.87 (8)		-0.009		0.002		0.001
M3−M2		20.75 (21)		0.008		-0.004		-0.009
Insomnia condition on ESS								
M1: Configural	93.65 (20)^a^		0.972		0.047		0.051	
M2: Plus all loadings constrained	105.34 (28)^a^		0.975		0.044		0.049	
M3: Plus all intercepts constrained	118.21 (36)^a^		0.980		0.041		0.046	
M2−M1		11.69 (8)		0.003		0.003		-0.002
M3−M2		12.66 (8)		-0.005		-0.003		-0.003

Abbreviations: CFI; comparative fit index; SRMR; standardized root mean square residual; RMSEA, root mean square error of approximation. M1 = Model 1, a configural model; M2 = Model 2, a model based on M1 with all factor loadings constrained being equal across groups; M3 = Model 3, a model based on M2 with all item intercepts constrained being equal across groups. ^a^ *P* < 0.05.

**Table 5:** Concurrent validity of the Epworth Sleepiness Scale for Children using regression model

**Criterion**	**B (SE)**	**β**	**95% CI**
Insomnia^a^	0.30 (0.02)	0.39	0.26-0.35
Age	0.15 (0.07)	0.06	0.01-0.31
Gender (Female)	-0.06 (0.25)	-0.01	-0.56-0.44
Father’s education	-0.10 (0.26)	-0.01	-0.61-0.40

^a^ Insomnia was measured using Insomnia Severity Index.

**Table 6:** Latent Class analysis to identify subgroups of adolescents

**Profile solution**	**AIC**	**BIC**	**SSABIC**	**Entropy**	**L-M-R test (** ***P *** **value)**
2	22379.648	22697.733	22503.961	0.801	1845.806 (<0.001)
3	21886.251	22365.985	22073.740	0.813	552.925 (0.537)
4	21706.708	22348.092	21957.372	0.824	240.468 (0.097)

Abbreviations: AIC, Akaika’s information criterion; BIC, Bayesian information criterion; SSABIC, sample-size adjusted BIC; L-M-R test, Lo-Mendell-Rubin’s likelihood ratio test.

**Table 7:** Comparisons among two subgroups of adolescents in different the daytime Sleepiness class

	**Low-risk Daytime sleepiness (n=595)**	**High-risk Daytime sleepiness (n=771)**	**Overall test** ***P *** **value**
Age in year, mean (SE)	15.37 (0.06)	15.64 (0.07)	0.002
Gender (female%)^a^	20.46 (0.01)	28.45 (0.01)	<0.001
ISI total score	14.93(0.21)	16.28(0.23)	0.002
Father’s education, mean (SE)	5.65 (0.04)	7.86 (0.04)	0.001
ESS total score	7.48(0.16)	8.48 (0.20)	<0.001

Abbreviations: ISI, Insomnia Severity Index; ESS, Epworth Sleepiness Scale. ^a^ *P* < 0.001.
